# Nitrogen and Sulfur Additions Improved the Diversity of *nirK*- and *nirS*-Type Denitrifying Bacterial Communities of Farmland Soil

**DOI:** 10.3390/biology10111191

**Published:** 2021-11-16

**Authors:** Xuan Chen, Hui Wei, Jiaen Zhang

**Affiliations:** 1 College of Natural Resources and Environment, South China Agricultural University, Guangzhou 510642, China; xuanchen@stu.scau.edu.cn (X.C.); weihui@scau.edu.cn (H.W.); 2 Guangdong Engineering Research Center for Modern Eco-Agriculture and Circular Agriculture, South China Agricultural University, Guangzhou 510642, China; 3 Guangdong Provincial Key Laboratory of Eco-Circular Agriculture, South China Agricultural University, Guangzhou 510642, China; 4 Key Laboratory of Agro-Environment in the Tropics, Ministry of Agriculture and Rural Affairs, South China Agricultural University, Guangzhou 510642, China; 5 Guangdong Laboratory for Lingnan Modern Agriculture, South China Agricultural University, Guangzhou 510642, China

**Keywords:** N and S addition, denitrification, abundance, diversity, community structure

## Abstract

**Simple Summary:**

In this study, we investigated the effects of combined N and S additions on soil properties and N-cycling microorganisms. We found that soil treated with N and S additions had higher Shannon diversity and Chao 1 richness for the *nirK*- and *nirS*-related OTUs. We also found that, in the *nirK*-type denitrifying community, the addition of N and S promoted increases in the relative abundance of *Bradyrhizobium*, and in terms of the *nirS*-type denitrifying community, the N and S additions increased the relative abundance of *Thiobacillus*. Moreover, the N and S additions increased the activity of nitrate reductase and nitrite reductase. Most importantly, the *nirK*-type denitrifying community demonstrated a higher sensitivity to N and S additions. We expect that this research will be useful for the study of the nitrogen cycle in soil.

**Abstract:**

Anthropogenic nitrogen (N) and sulfur (S) deposition can change above- and belowground biodiversity, including soil microbial diversity. The diversity of denitrifying microorganisms is of great significance to the calculation of the global nitrogen cycle and nitrogen flux. For a long time, *nirK* and *nirS* have been used as the functional genes to study denitrifying microorganisms, and have gradually become molecular markers for studying the composition and diversity of denitrifying bacteria. Here, three-time exposures to N and S applications (7, 30, and 60 days), were independently established. Additionally, the abundance, diversity, and structure of *nirK*- and *nirS*-type denitrifying communities were examined by sequencing analyses in response to three treatments, namely, N and S (T_N/S_), sodium chloride (T_NaCl_) and deionized water (pH = 7.0) (CK). Our results suggest that T_N/S_ led to higher electrical conductivity (EC), total nitrogen (TN), total organic carbon (TOC), nitrate nitrogen (NO_3_^−^-N), ammonium nitrogen (NH_4_^+^-N), and lower pH compared with T_NaCl_ and CK, which affected the diversity of *nirK*- and *nirS*-type denitrifying bacterial communities. We also observed that the *nirK*-type denitrifying community demonstrated a higher sensitivity to N and S additions. Overall, our results are important for the understanding of nitrogen in soil and N_2_O emissions.

## 1. Introduction

In recent years, the rapid development of industry and agriculture has led to an increase in global atmospheric N deposition [1,2,3,4,5,6,7]. Over the past two centuries, anthropogenic N input in ecosystems has increased by nearly 10 times [8], and it is expected to double by 2050 [2]. The imbalance of the natural N cycle has become increasingly serious. Overall, the impact of N deposition exists on a global scale and has a profound impact on ecosystems [9,10], especially on terrestrial ecosystems [11,12]. At the same time, N deposition also affects other ecological types, such as oceans [13], lakes [14], and seaport systems [15]. The dramatic increase in N deposition leads to changes in the N cycle [16], which has adverse effects on ecosystems, such as the eutrophication and acidification of ecosystems [17], and changes in microbial community structures and diversity in soils [18].

Soil microorganisms are important parts of the ecosystem [19], and they play an important role in material circulation, litter decomposition, and maintaining the stability of the ecosystem [20,21]. Therefore, it is valuable to explore the effect of N deposition on soil microorganisms. The current research on the effects of N deposition on soil microorganisms mainly focuses on microbial biomass, community structure and diversity, and microbial functional diversity. Studies have shown that the community structure and diversity of soil microorganisms respond differently under different N deposition conditions. Sui et al. [22] found that N deposition increased the diversity of bacteria in the Sanjiang Plain, and that the bacterial diversity was the highest when the level of N was low, indicating that the impact of N deposition on the increase in bacterial diversity had a certain concentration limit. The addition of N can also change the abundance of different phyla of soil microorganisms. Fierer et al. [23] and Ramirez et al. [24] found that copiotrophic taxa (including members of the *Proteobacteria* and *Bacteroidetes phyla*) typically increased in relative abundance in the high-N plots, with oligotrophic taxa (*Acidobacteria* and *Verrucomicrobia*) exhibiting the opposite pattern. Moreover, Cederlund et al. [25] have shown that N fertilization reduced the relative abundances of *Acidobacteria*, *Gemmatimonadetes*, and *Verrucomicrobia*. In addition, other studies have shown that N deposition affects the diversity of microbial functional genes by changing soil microbial activity. Yu et al. [26] reported that for the soil microbial gram aerobic bacteria, the metabolic function was enhanced significantly under different N additions, while the metabolic activity of soil microbial fungi and yeast was unaffected by different N additions. Wu et al. [27] also found that N deposition enhanced the metabolic function of microorganisms.

Sulfur, another nutrient element, also plays an important role in the nutrient cycle of the ecosystem. However, atmospheric sulfur deposition is the main factor in soil acidification in terrestrial ecosystems, which causes the release of base ions, nutrient imbalance, and has an impact on the structure and function of soil biological communities [28]. Studies have shown that soil acidification not only reduces the number of microorganisms but also inhibits their growth and activity [29,30]. Wang et al. [31] confirmed that *Actinomycetes* and *Bacteria* were greatly reduced and their activity was decreased due to soil acidification, while fungi were less affected by soil acidification. Acidification also affects the reproduction of microbial populations. Sierra et al. [32] indicated that it is not conducive to the survival of *Pseudomonas* and *Corynebacterium*. In addition, Wang et al. [33] found that the relative abundance of *Lysobacter* and *Flavobacterium* was significantly increased, while the relative abundance of *Massilia* was significantly reduced. Overall, high levels of N and S emissions have caused a large amount of acid deposition, but previous studies paid more attention to the negative effects of acid rain, while potential fertilization effects of acid rain-induced N and S inputs have rarely been studied.

Denitrification is an important link in the complete cycle of nitrogen [34]. Heterotrophic denitrification comprises sequential reduction steps from NO_3_^−^ to NO_2_^−^, NO, N_2_O, and finally to elemental N_2_ [35]. Specific enzymes, namely NO_3_^−^ reductase (Nar), NO_2_^−^ reductase (Nir), NO reductase (Nor), and N_2_O reductase (Nos) regulate the rate and product stoichiometry of heterotrophic denitrification [36,37]. From an ecological point of view, the denitrification process is a self-balancing mechanism that can protect the system from instability and imbalance [38]. Moreover, denitrification is one of the main pathways of nitrogen loss [39]. Marcel et al. [40] showed that nitrogen which was recycled through denitrification accounted for 52% of the total global nitrogen input to the soil, while the denitrification loss of nitrogen fertilizer accounted for 12% of nitrogen fertilizer applications [41]. In addition, denitrification is the main source of greenhouse gases, such as N_2_O [42]. In 1990, the application of chemical nitrogen fertilizer resulted in about 1.5 × 10^6^ t N_2_O-N, accounting for 44% of the N_2_O-N release into the atmosphere by human activities [3], but by 2020, the figure in China had reached 4.30 times that of 1990 [42]. Overall, the denitrification process has a significant impact on the nitrogen cycle, fertilizer loss, and the environment. In-depth research into this is helpful for us to further quantify nitrogen turnover, predict the fate of nitrogen fertilizer in farmland ecosystems, formulate sewage treatment measures, and deal with global warming caused by the greenhouse effect.

**Figure 1:**
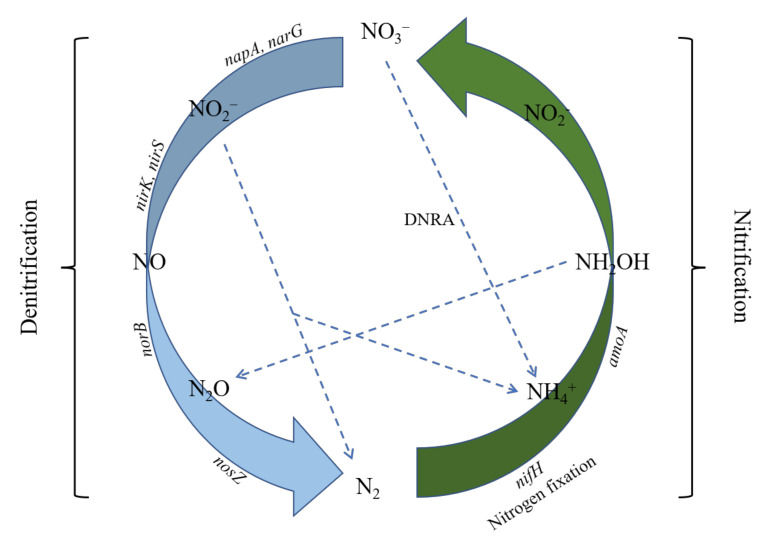
Functional genes for nitrification and denitrification. Abbreviations: NO: nitric oxide; NO_2_^−^: nitrite ion; NO_3_^−^: nitrate ion; NH_4_^+^: ammonium ion; NH_2_OH: hydroxylamine; N_2_: nitrogen; DNRA: dissimilatory nitrate reduction to ammonium. Dotted lines indicate possible reaction process.

The reduction of nitrite to NO is the most important step in denitrification (Figure 1), which is catalyzed by Nir. There are two types of enzymes involved in this process. One is the soluble copper-containing enzyme (Cu-Nir), which is encoded by the *nirK* gene. The other is cytochrome reductase (cd1-Nir), which is encoded by the *nirS* gene. These two enzymes perform the same function, but feature different structures and catalytic sites and generally do not exist in the same denitrification species at the same time. They represent two denitrification groups with different ecological characteristics and occupy different niches [43]. Generally, *nirS*-type denitrifying bacteria feature higher gene abundance and dominate the environment, while *nirK*-type denitrifying bacteria exist in a wider microbial population and cover more diverse taxonomic units [39]. Studies have shown that *nirK*-type denitrifying bacteria exhibit more powerful habitat selection in response to different environmental gradients [43,44]. *NirK* and *nirS* were the first functional genes used to study denitrifying microorganisms [45], and have gradually become molecular markers for studying the composition and diversity of denitrifying bacteria. Studies have shown that nitrogen addition significantly increased the abundance of the NO_2_^−^ reducing genes (*nirK* and *nirS*) in Chinese fir plantations [46]. There is also a study indicating that long-term fertilizer applications had a significant impact on the size of the *nirK* community, but had little impact on the *nirS* community [47]. Previous studies have shown that N deposition levels [48] and N forms [49] can affect the abundance and community compositions of N-cycle functional genes, whereas few studies have addressed the influence of S additions and, in particular, the interaction between S and N on the paddy soil microbial communities involved in N cycling. Therefore, it is necessary to better understand how mixed additions of N and S influence the N cycling process in soil ecosystems.

Additionally, in complex and variable soil environments, the coexistence of *nirS*- and *nirK*-type denitrifying communities leads to cooperation and competition, which are factors determining community variation [50,51]. Therefore, in this regard, the potential ecological roles of microorganisms may be revealed by the network analyses used to study the co-existence within functional groups [52,53]. However, for now, few such studies have focused on functionally equivalent genes [51]. Thus, the study of functional equivalent genes may help to improve our understanding of soil denitrifying microbial communities.

In this study, we investigated the effects of combined N and S additions on soil properties and N-cycling microorganisms. Based on the molar ratio of S to N in acid rain of 2.75:1 [54], we conducted this experiment to study how N and S additions affect denitrifying microbial communities and enzyme activities in the paddy soil. We hypothesized that: (1) the soil treated with N and S additions would have higher Shannon diversity and Chao 1 richness for *nirK*- and *nirS*-related operational taxonomic units (OTUs); (2) at the genus level, in the case of the *nirK*-type denitrifying community, the application of N and S could promote increases in the relative abundance of *Bradyrhizobium*, while in the *nirS*-type denitrifying community, the application of N and S might increase the relative abundance of *Thiobacillus*; (3) N and S additions would increase the activity of nitrate reductase and nitrite reductase. We expected that we could use this study to better evaluate the impact of N and S deposition on ecosystems, and that it might provide an effective reference for the study of the nitrogen cycle.

## 2. Materials and Methods

### 2.1. Site Description and Experimental Design

The experimental soils were collected from an ecological farm (23°06′ N, 113°20′ E) of South China Agricultural University in Guangzhou City, Guangdong Province, China. The climate in this area is classified as a subtropical monsoon climate, according to the Köppen–Geiger climate classification. In addition, the average annual air temperature and precipitation are 22 °C and 1800 mm, respectively. The soil is classified as Rhodudult [55]. The soil pH was 6.2, with an organic matter content of 19.64 g·kg^−1^, a TN of 0.98 g·kg^−1^, and an EC of 73.33 μS·cm^−1^. Furthermore, the other chemical properties of the soil, including contents of the available phosphorous (AP), the available potassium (AK), NH_4_^+^-N, and NO_3_^−^-N, were 40.88, 144.76, 10.70, and 28.82 mg·kg^−1^, respectively.

Three-time exposures to N and S applications (7, 30, and 60 days) were independently established, and three treatments with five replications of each were set up, including T_N/S_, T_NaCl_, and CK. Among them, CK was set as the control. N was added as sodium nitrate (NaNO_3_) and S was added as sodium sulfate (Na_2_SO_4_). To observe the effects of Na input, we set up an extra T_NaCl_ treatment with an input of the same amount of Na as in the T_N/S_ treatment. NaNO_3_, Na_2_SO_4,_ and NaCl were purchased from Guangzhou Congyuan Instrument Ltd., Guangzhou, China. According to the characteristics of the N and S input associated with acid rain in Guangzhou, a mixed solution of NaNO_3_ and Na_2_SO_4_ was prepared with a molar ratio of S to N of 2.75:1 [54], that is, the concentrations of NaNO_3_ and Na_2_SO_4_ were 0.60 mol·L^−1^ and 1.06 mol·L^−1^, respectively, and they were sealed and stored for reserve. Before the experiment began, each conical flask (200 mL) was filled with 150 g of soil and 27 mL of treatment solution, respectively. All the soil samples were placed in a temperature-controlled (28 °C) incubator (RXZ-500A) in the dark and the soil moisture was controlled at 60% of the water holding capacity throughout the incubation period. At the end of each incubation time, 3 g of soil from each sample were stored at −20 °C for DNA extraction and the remaining sample was kept at 4 °C for further analysis of the soil’s physicochemical properties and enzyme activities.

### 2.2. Soil Sampling and Physicochemical Analysis

The NO_3_^−^-N and NH_4_^+^-N were measured using the methods described by Bao [56]. The EC and pH were measured in a 1:5 (*w*:*v*) aqueous extract, with a conductivity meter and pH meter (Crison mod. 2001, Barcelona, Spain), respectively. The TN and TOC concentrations were determined using an elemental analyzer (Vario TOC cube). The nitrate reductase assays were carried out according to the method described by Li et al. [57]. One unit of enzyme activity (U) was defined as the number of milligrams of nitrite produced per g of soil sample in 24 h at 30 °C. The nitrite reductase assays were determined by the Griess reagent colorimetric method [58]. One unit of enzyme activity (U) was defined as the number of milligrams of the difference between the content of nitrite nitrogen before and after the enzymatic reaction in per g of soil sample in 24 h at 30 °C.

### 2.3. Microbial DNA Extraction, nirK, and nirS Genes Amplification and Sequencing

The batch tests samples obtained on days 7, 30, and 60 were used for DNA extraction in triplicate with the OMEGA soil DNA extraction kit (USA), following the manufacturer’s instructions. This study used 1% agarose gel electrophoresis to determine the quality and concentration of the extracted DNA, which was finally checked with a NanoDrop ND-2000 spectrophotometer (NanoDrop Technologies, Wilmington, DE, USA) and stored at −20 °C until use. The primers nirK1aCuF (ATCATGGTSCTGCCGCG) and nirKR3CuR (GCCTCGATCAGRTTGTGGTT) were used to amplify the hypervariable region fragments of *nirK* genes [59], and the primers cd3aF (GTSAACGTSAAGGARACSGG) and R3cdR (GASTTCGGRTGSGTCTTGA) were used to amplify the hypervariable region fragments of *nirS* genes [57]. The 20-μL PCR reaction consisted of a 4 μL of 5 × FastPfu Buffer, 2 μL of 2.5 mmol·L dNTPs, 0.8 μL of each primer (5 μM, Forward and Reverse), 0.4 μL of FastPfu Polymerase, 0.2 μL of BSA and 10 ng of template DNA. The thermal cycling programs were as follows: initial denaturation at 95 °C for 3 min; 40 cycles of 95 °C for 30 s, 60 °C for 30 s, 72 °C for 45 s; and a final extension at 72 °C for 10 min. The PCR products were purified using an AxyPrep DNA Gel Extraction Kit (Axygen Bio-Sciences, Union City, CA, USA), and then the purified products of *nirS* and *nirK* were pooled in equimolar quantities. Finally, according to the standard protocols issued by Majorbio Bio-pharm Technology Co., Ltd. (Shanghai, China), paired-end sequencing (2 × 250) was performed using the Illumina MiSeq platform (Illumina, San Diego, CA, USA). QIIME was used to filter the raw data and screen out sequences shorter than 150 bp, low-quality sequences, and mismatched sequences, and the filtered data were normalized at the same depth by Mothur software. In addition, through the UPARSE pipeline 7.1 [60], all the optimized sequences were clustered into OTUs according to the principle of 97% similarity clustering. An average of 194,400 *nirK* and 214,806 *nirS* effective sequence reads were obtained from each sample, and the average lengths of *nirK* and *nirS* were 450 bp and 400 bp, separately.

### 2.4. Statistical Analysis

According to the identification results of the OTU division and classification level, the specific composition of each sample at each classification level (kingdom, phylum, class, order, family, genus, species) was obtained by QIIME version 1.8.0. Moreover, two α diversity indices (Chao 1 index and Shannon index) for each sample were calculated by QIIME version 1.8.0. One-way ANOVA was performed using SPSS16.0. The similarity analysis (ANOSIM) and non-metric multidimensional scaling (NMDS: based on Bray–Curtis distance) of the *nirK*- and *nirS*-type denitrifying communities between different treatments were carried out through the “Vegan” package, and the data were visualized by the “ggplot2” package in R software (Version 4.0.5). In addition, CANNOCO 5.0 software was used for redundancy analysis (RDA) of dominant denitrifying microorganisms and soil physicochemical properties. Meanwhile, the Spearman test was used to analyze the correlation between them, and the correlation heat map was drawn by using R software.

## 3. Results

### 3.1. Physico-Chemical Analysis of Soil Samples

Relative to the T_NaCl_ and CK, we found that the soil physicochemical properties changed significantly at 7-day incubation and 60-day incubation in the T_N/S_ (Table 1), including significant increases in the EC, TN, TOC, NO_3_^−^-N, and NH_4_^+^-N, and a significant reduction in the pH (*p* < 0.05). The 30-day incubation did not significantly change the investigated soil physicochemical properties (*p* > 0.05, Table 1).

### 3.2. Alpha-Diversity Indices of nirS- and nirK-Type Denitrifying Communities

**Figure 2:**
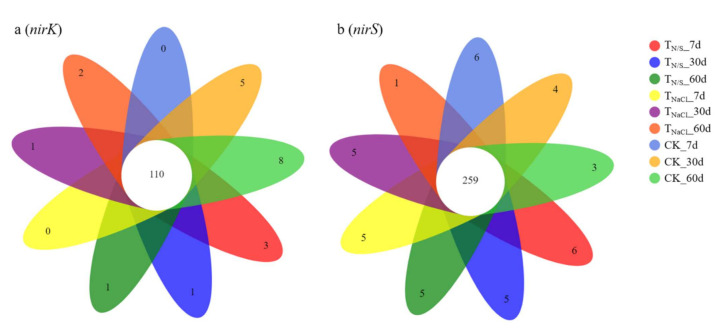
Venn diagram of sample OTUs quantity. Different numbers after the treatments indicate the treatment duration. Lower-case letters (a and b) represent different genes. Treatments: T_N/S_: NaNO_3_ + Na_2_SO_4_; T_NaCl_: NaCl; CK: control. There were 110 OTUs shared by all groups (**a**). There were 259 OTUs common to all groups (**b**).

**Figure 3:**
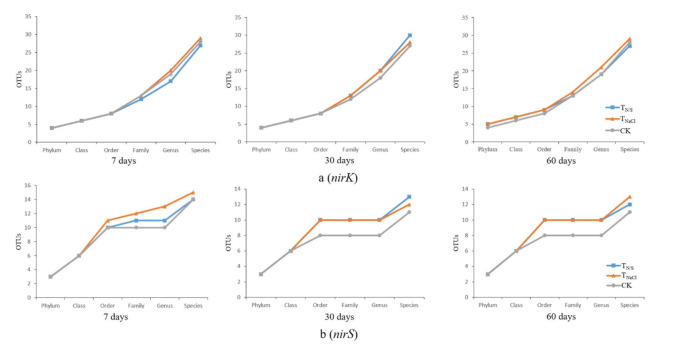
The number of OTUs of the sample at different classification levels. Lower-case letters (a and b) represent different genes. Treatments: T_N/S_: NaNO_3_ + Na_2_SO_4_; T_NaCl_: NaCl; CK: control. In terms of the nirK gene, on the 7th day, the numbers of OTUs at the phylum, class, and order levels under each treatment were the same, while the numbers of OTUs at the family, genus, and species levels were lower than those of the control, while after 30, 60 days of incubation, the numbers of OTUs in the soil treated with TN/S at the fam-ily, genus, and species levels were higher than those of the control (**a**). In terms of the nirS gene, we found that there was a non-significant difference in the number of OTUs between the treatments at the phylum and class levels. However, a significant difference was observed, that is, compared with CK, TN/S increased the numbers of OTUs at the order, family, genus, and species levels in all three time periods (**b**).

In terms of the *nirK* gene, a total of 312 OTUs for species classification were obtained by clustering. On the 7th day, the total OTUs of T_N/S_, T_NaCl_, and CK were 191, 203, and 213, respectively. Similarly, on the 30th day and the 60th day, they were 221, 210, and 220 and 197, 219, and 228, respectively. Among them, there were 110 OTUs shared by all groups (Figure 2a). As for the *nirS* gene, a total of 751 OTUs for species classification were obtained by clustering. With seven-day incubation, the total OTUs of T_N/S_, T_NaCl,_ and CK were 529, 490, and 561, respectively. Similarly, they were 533, 513, and 493 and 508, 508, and 513 with 30-day incubation and 60-day incubation, respectively. Of these, there were 259 OTUs common to all groups (Figure 2b). After OTU identification, the OTUs of each sample were divided into six classification levels, including phylum, class, order, family, genus, and species. The results showed that the *nirK*-type denitrifying microbial communities were more abundant. In terms of the *nirK* gene, on the 7th day, the numbers of OTUs at the phylum, class, and order levels under each treatment were the same, while the numbers of OTUs at the family, genus, and species levels were lower than those of the control, while after 30, 60 days of incubation, the numbers of OTUs in the soil treated with T_N/S_ at the family, genus, and species levels were higher than those of the control (Figure 3a). In terms of the *nirS* gene, we found that there was a non-significant difference in the number of OTUs between the treatments at the phylum and class levels. However, a significant difference was observed, that is, compared with CK, T_N/S_ increased the numbers of OTUs at the order, family, genus, and species levels in all three time periods (Figure 3b).

Compared with CK, T_N/S_ resulted in a significantly higher Shannon diversity of *nirS*-related OTUs at 30-day incubation and 60-day incubation, and a significantly lower Chao 1 richness at 60-day incubation. Conversely, the T_N/S_-treated soils demonstrated higher Shannon diversity and Chao 1 richness for *nirK*-related OTUs relative to that of T_NaCl_ and CK throughout the incubation period (Table 2).

### 3.3. Beta Diversity of nirS- and nirK-Type Denitrifying Microorganisms

**Figure 4:**
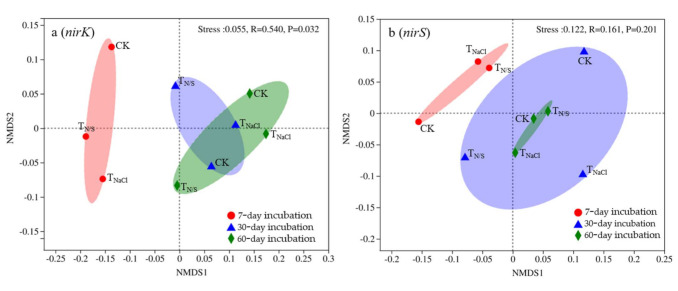
Non-metric multidimensional scaling analysis of *nirK*- (**a**) and *nirS*-type (**b**) denitrifying communities under different treatments. Treatments: T_N/S_: NaNO_3_ + Na_2_SO_4_; T_NaCl_: NaCl; CK: control.

NMDS analysis showed that the *nirK*-type community at 7-day incubation was separated from those in the other two periods (30-day incubation and 60-day incubation) (ANOSIM *R* = 0.540, *p* = 0.032) (Figure 4a). Moreover, in the *nirS*-type denitrifying community, we found that the microbial community changed significantly from the 7th day to the 60th day, and the similarity of the microbial community changed from small to large and then to small in different periods. However, such shifts were not statistically significant (ANOSIM *R* = 0.161, *p* = 0.201) (Figure 4b). Interestingly, we found a significant similarity in the community composition of soil *nirS* denitrifying microorganisms between T_N/S_ and T_NaCl_ at 7-day incubation. We also found a similarity between T_N/S_ and CK at 60-day incubation, while there was a significant difference in the community composition of soil *nirK* denitrifying microorganisms between each treatment at each stage. The results indicated that treatment duration could affect the community composition of denitrifying microorganisms, and the *nirK*-type denitrifying community was more responsive to treatment duration.

**Figure 5:**
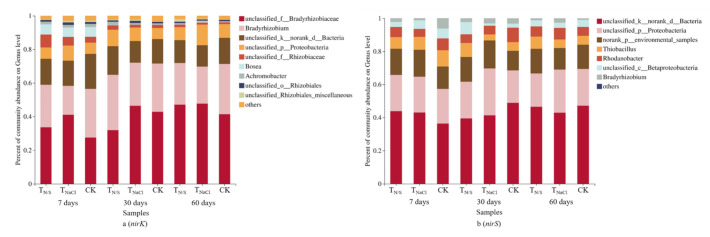
The community compositions of *nirK*- (**a**) and *nirS*-type (**b**) denitrifying communities under different treatments. Treatments: T_N/S_: NaNO_3_ + Na_2_SO_4_; T_NaCl_: NaCl; CK: control. At the genus level, a large number of *nirK* reads (0.3–20.8%) and *nirS* reads (1.4–48.8%) are defined as “unidentified” OTUs, suggesting that there remains a large degree of *nirK* and *nirS* diversity, which is not reflected in the current genome database [61].

Under different treatment conditions, the application of N and S changed the compositions of the *nirK*- and *nirS*-type denitrifying communities. At the genus level, *Bradyrhizobium* was the dominant *nirK* genus, accounting for 17.2–32.9% of the total *nirK*-type denitrifying community sequences in all soil samples (Figure 5a). Compared with T_NaCl_ and CK, we found that in the T_N/S_ treatment groups, the relative abundance of *Bradyrhizobium* significantly increased at 30-day incubation, but reduced at 60-day incubation. In the *nirS*-type denitrifying community, we observed that the predominant genus was *Thiobacillus*, accounting for 3.6–9.8% of the total *nirS* sequences (Figure 5b). Compared with the other two treatments, it is evident that the composition of the *nirS*-type denitrifying community was not sensitive to T_N/S_.

### 3.4. Enzyme Activities of Soil Samples

As expected, the nitrate reductase and the nitrite reductase were affected significantly by N and S additions (*p* < 0.05, Table 3), especially at 7-day incubation. We found that in the case of the nitrate reductase, it increased by 6.63% and 6.20%, respectively (*p* < 0.05), during that period when compared to the T_NaCl_ and CK. Similarly, it increased by 34.71% and 37.98%, respectively (*p* < 0.05), over the same time for the nitrite reductase.

### 3.5. Correlations between Functional Genes and Soil Properties

**Figure 6:**
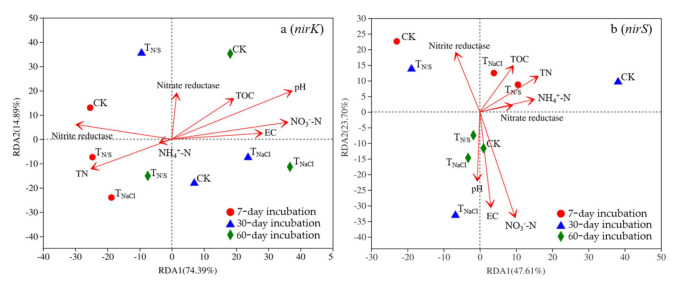
Redundancy analysis (RDA) of soil properties and functional gene abundances from different treatments. Lower-case letters (a and b) represent different genes. Treatments: T_N/S_: NaNO_3_ + Na_2_SO_4_; T_NaCl_: NaCl; CK: control. In the case of the nirK-type denitrifying communities, pH and NO_3_^−^-N were signifi-cantly positively correlated with axis 1, while nitrite reductase was significantly nega-tively correlated (**a**). Similarly, in the nirS-type denitrifying communities, TN and NH_4_^+^-N were significantly positively correlated with axis 1, while nitrite reductase was significantly negatively correlated (**b**).

A redundancy analysis (RDA) was used to explore the environmental factors affecting the composition of *nirK*- and *nirS*-type denitrifying microbial communities (Figure 6), and eight soil physical and chemical factors (pH, EC, TOC, NO_3_^−^N, and NH_4_^+^-N, nitrate reductase, nitrite reductase) affecting the microbial community were selected and analyzed with an OTUs table. In the case of the *nirK*-type denitrifying communities, pH and NO_3_^−^-N were significantly positively correlated with axis 1, while nitrite reductase was significantly negatively correlated (Figure 6a). In addition, according to the projection of physicochemical factors on the first axis, pH may have been the first influencing factor. Similarly, in the *nirS*-type denitrifying communities, TN and NH_4_^+^-N were significantly positively correlated with axis 1, while nitrite reductase was significantly negatively correlated (Figure 6b). Moreover, TN may have been the first influencing factor through the projection of physicochemical factors on the first axis.

**Figure 7:**
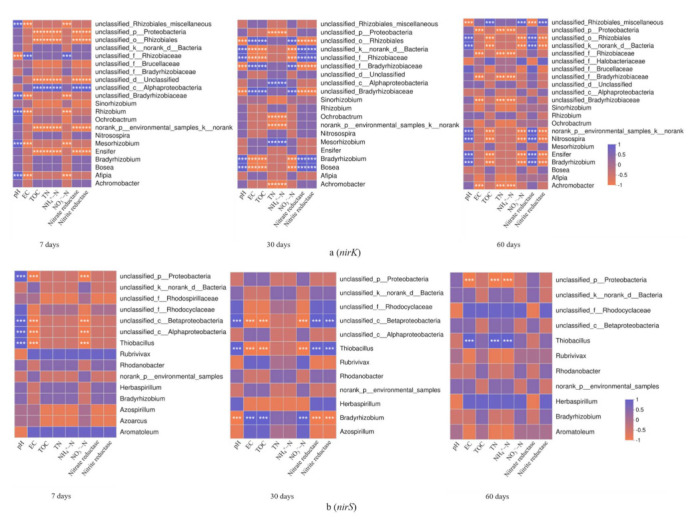
Correlation heatmap of soil physicochemical factors and *nirK*- (**a**) and *nirS*-type (**b**) denitrifying communities in different periods. The *R* values are shown in different colors, and *** indicates *p* ≤ 0.001.

In order to select the 50 most abundant bacterial genera, the Spearman correlation coefficient was used to analyze the correlation between soil physicochemical factors and the composition of soil *nirK*- and *nirS*-type denitrifying microbial communities in the three treatments (Figure 7). The results showed that there were differences in the correlation between the two genotypes of denitrifying microbial communities and soil physicochemical factors, but the difference was more obvious in the *nirK* genes. In the case of the *nirK*-type denitrifying communities, we found that only two genera were significantly associated with pH at 7-day incubation, namely *Mesorhizobium* and *Afipia* (Figure 7a) (*p* ≤ 0.001). At 30-day incubation, three genera were significantly related to pH, TN, NH_4_^+^-N, nitrate reductase, and nitrite reductase, respectively, namely *Mesorhizobium*, *Bradyrhizobium*, and *Bosea* (Figure 7a) (*p* ≤ 0.001); while at the 60-day incubation, four genera were significantly related to pH and nitrate reductase, respectively, namely *Norank_p_environmental_samples_k_norank*, *Nitrosopira*, *Ensifer*, and *Bradyrhizobium* (Figure 7a) (*p* ≤ 0.001). In terms of the *nirS*-type denitrifying communities, *Thiobacillus* was only significantly associated with pH at 7-day incubation. However, at 30-day incubation and 60-day incubation, *Thiobacillus* was also related to nitrate reductase, nitrite reductase, and EC, TN, NH_4_^+^-N, respectively (Figure 7b) (*p* ≤ 0.001).

## 4. Discussion

### 4.1. Impact of N and S Additions on the Community Structure and Diversity of Soil nirK- and nirS-Type Denitrifying Microorganisms

Denitrifying microorganisms widely exist among bacteria and archaea, and denitrification is also found in some fungal mitochondria [37]. In this study, N and S applications were found to have a significant effect on soil properties, which in turn contributed to promoting changes in the abundance and community compositions of *nirK*- and *nirS*-type denitrifying bacteria. Through the present research, we found that the application of N and S increased the content of TOC in the soil, which provided essential nutrients for the increase of bacterial biomass [62]. Therefore, we found that bacteria were mainly involved in denitrification in the three treatments, and many unidentified gene sequences were also found. As for whether there are denitrifying fungi in the soil, further analysis and the identification of the *nirK* and *nirS* gene sequences of these unknown species are also needed. Among the identified *nirK* and *nirS* denitrifying microorganisms, *Proteobacteria* was the most prominent among the three treatments, and fertilization significantly increased its relative abundance. It was confirmed that *Proteobacteria* can use difficult biodegradable carbon sources in acidic environments and decompose them into small molecular substances to provide nutrients for other microorganisms [63]. This is consistent with the results of this study. N and S applications decreased the pH of the soil, which created a favorable acidic environment for *Proteobacteria*. Thus, the abundance of *Proteobacteria* was higher under fertilization. It is worth noting that the abundance of the *nirK* gene was significantly correlated with soil pH. As mentioned above, soil pH is a key factor affecting the responses of denitrifier genes to N fertilization, which may be because pH is closely related to the metabolic substrate content of dissolved organic carbon and nitrate [49,64,65,66] and directly or indirectly affects denitrifying communities. Other associated factors include NO_3_^−^-N. As one of the initial substrates of denitrification, NO_3_^−^-N is an important factor in controlling the denitrification of *nirK*-type denitrifying bacteria [67]. At the genus level, in terms of the *nirK* gene, our results demonstrated that *Bradyrhizobium* was the core genus, which was consistent with the results of Fan et al. [68]. Previous studies reported that the dominant genera, including *Bradyrhizobium* and *Burkholderia* were facultative nitrogen-fixing bacteria, which were found in *Alpha-Proteobacteria* and *Beta-Proteobacteria*, respectively, and they were usually classified as eutrophic organisms. These advantageous eutrophic organisms can use large amounts of inorganic substances, such as NO_3_^−^-N, as energy sources to reproduce rapidly in a nutrient-rich environment [69,70,71]. The results of this study also supported the hypothesis that the application of N increased the content of NO_3_^−^-N in the soil compared with the other two treatments, which were more conducive to the growth of *Bradyrhizobium*. As for the *nirS* gene, our results indicated that *Thiobacillus*, a typical sulfur-oxidizing bacteria, was the dominant genus. In our study, the addition of S led to an increase in the SO_4_^2−^ content in the soil, with nitrite gradually replaced by sulfate, resulting in a significant increase in the relative abundance of *Thiobacillus* [72]. Additionally, in our study, TN and NH_4_^+^-N were significantly related to *nirS*, but not correlated with *nirK* denitrifying bacteria. Previous studies demonstrated that the composition and abundance of denitrifying bacteria could be affected by indirectly creating an environment favorable to denitrifying bacteria [49,73,74,75]. Other studies also reported that the abundance and community structure of soil bacteria in farmland were affected by many factors. Different water management systems caused differences in pH, inorganic nitrogen content, microbial biomass carbon content, microbial biomass nitrogen content, soluble organic nitrogen content, and soluble organic carbon content. All of these factors may lead to differences in soil bacterial abundance and community structure [76,77,78,79].

The results of this study indicated that the addition of N and S significantly increased the alpha diversity index of *nirK*-type denitrifying communities, while the *nirS*-OTU-related Shannon and Chao 1 indices were less affected, suggesting that alpha diversity was not necessarily affected by the change in population sizes and compositions of denitrifying communities [80,81]. In conclusion, compared with the change in *nirS*-type denitrifying communities at 60-day incubation, the *nirK*-type denitrifying communities responded more strongly to the application of N and S. These observations confirmed that *nirK*- and *nirS*-type denitrifying communities respond differently to N and S applications, and it is clear that the most sensitive is the *nirK*-type denitrifying community, as was revealed in previous studies [82,83].

### 4.2. Impact of Treatment Duration on the Diversity of Soil nirK- and nirS-Type Denitrifying Microorganisms

The NMDS analysis showed that treatment duration could affect the community composition of denitrifying microorganisms. This may be because with the increase in time, the contents of carbon and nitrogen change, which can affect the diversity of soil-denitrifying microorganisms [84]. On the other hand, pH is also one of the factors influencing microbial diversity [35,85]. When the soil pH value exceeds a certain range (niche), the net growth of a single taxon that cannot survive decreases, which may alter the competitive outcome [45,86]. However, in this study, the pH is within the optimal range of all microorganisms (5.9–6.8), so there are no extreme conditions. At present, there are few studies on the effect of treatment duration on the diversity of soil *nirK*- and *nirS*-type denitrifying substances, so further study is needed in the future.

## 5. Conclusions

To summarize, genes for denitrification are ubiquitous in soil microorganisms. The abundance of *nirK* and *nirS* genes was linked to soil pH, NO_3_^−^-N concentration, TN concentration, and NH_4_^+^-N concentration. Through the quantitation of *nirS* and *nirK*, the addition of N and S had an effect on soil denitrification. Therefore, further study of the application of N and S will contribute to our knowledge of how the soil community composition affects N_2_O flux. The difference between *nirK* and *nirS* abundance may provide a more accurate result than using single genes.

## Figures and Tables

**Table 1:** Physicochemical properties of soil samples.

Days	Treatments	pH	EC (μS·cm^−1^)	TN (g·kg^−1^)	TOC (g·kg^−1^)	NO_3_^−^-N (mg·kg^−1^)	NH_4_^+^-N (mg·kg^−1^)
7	T_N/S_	5.9 + 0.0 ^b^	115 + 8.9 ^a^	1.14 + 0.28 ^a^	28 + 0.6 ^a^	28 + 0.6 ^a^	11 + 0.2 ^a^
	T_NaCl_	6.2 + 0.0 ^a^	82 + 4.6 ^b^	1.03 + 0.02 ^b^	20 + 0.5 ^b^	17 + 0.1 ^b^	7 + 0.3 ^b^
	CK	6.2 + 0.1 ^a^	70 + 3.5 ^b^	1.05 + 0.04 ^b^	20 + 0.8 ^b^	16 + 0.4 ^c^	7 + 0.2 ^b^
30	T_N/S_	6.8 + 0.1 ^a^	128 + 7.5 ^a^	1.09 + 0.07 ^a^	22 + 0.3 ^a^	44 + 0.7 ^a^	14 + 0.6 ^a^
	T_NaCl_	6.7 + 0.1 ^a^	137 + 3.6 ^a^	1.11 + 0.08 ^a^	22 + 0.7 ^a^	45 + 0.9 ^a^	14 + 0.5 ^a^
	CK	6.8 + 0.1 ^a^	130 + 7.5 ^a^	1.15 + 0.09 ^a^	22 + 0.4 ^a^	45 + 0.6 ^a^	15 + 0.7 ^a^
60	T_N/S_	6.3 + 0.0 ^b^	266 + 2.1 ^a^	1.14 + 0.02 ^a^	23 + 0.6 ^a^	51 + 0.8 ^a^	6 + 0.1 ^a^
	T_NaCl_	6.8 + 0.0 ^a^	220 + 8.5 ^b^	0.90 + 0.02 ^b^	17 + 0.4 ^b^	48 + 0.6 ^b^	5 + 0.1 ^b^
	CK	6.9 + 0.1 ^a^	224 + 8.1 ^b^	0.96 + 0.03 ^c^	17 + 0.1 ^b^	48 + 0.5 ^b^	5 + 0.0 ^b^

All values are presented as mean ± standard error (*n* = 5); different letters in the same column under the same period indicate significant differences between treatments (*p* < 0.05). Treatments: T_N/S_: NaNO_3_ + Na_2_SO_4_; T_NaCl_: NaCl; CK: control.

**Table 2:** Alpha diversity index of soil *nirK*- and *nirS*-type denitrifying microorganisms.

Gene Types	Days	Sample	Shannon	Chao 1
*nirK*	7	T_N/S_	2.77 ± 0.01 ^a^	246 ± 1.8 ^a^
		T_NaCl_	2.56 ± 0.00 ^c^	219 ± 4.4 ^c^
		CK	2.73 ± 0.00 ^b^	237 ± 5.4 ^b^
	30	T_N/S_	3.38 ± 0.01 ^a^	274 ± 8.5 ^a^
		T_NaCl_	3.07 ± 0.01 ^b^	242 ± 10.8 ^b^
		CK	3.03 ± 0.01 ^c^	238 ± 8.4 ^b^
	60	T_N/S_	3.07 ± 0.01 ^a^	262 ± 3.0 ^a^
		T_NaCl_	2.79 ± 0.01 ^c^	254 ± 5.3 ^b^
		CK	3.03 ± 0.01 ^b^	228 ± 2.7 ^c^
*nirS*	7	T_N/S_	4.49 ± 0.01 ^b^	619 ± 4.8 ^a^
		T_NaCl_	4.34 ± 0.00 ^c^	620 ± 9.6 ^a^
		CK	4.51 ± 0.01 ^a^	620 ± 12.4 ^a^
	30	T_N/S_	4.60 ± 0.01 ^a^	619 ± 9.2 ^a^
		T_NaCl_	4.61 ± 0.01 ^a^	623 ± 13.1 ^a^
		CK	4.55 ± 0.00 ^b^	619 ± 7.2 ^a^
	60	T_N/S_	4.53 ± 0.01 ^a^	602 ± 11.2 ^b^
		T_NaCl_	4.54 ± 0.01 ^a^	596 ± 7.3 ^b^
		CK	4.51 ± 0.00 ^b^	615 ± 7.7 ^a^

All values are presented as mean ± standard error (*n* = 5); different letters in the same column under the same gene and the same period indicate significant differences between treatments (*p* < 0.05). Treatments: T_N/S_: NaNO_3_ + Na_2_SO_4_; T_NaCl_: NaCl; CK: control.

**Table 3:** Enzyme activities of soil samples.

Enzymes	Treatments	7 days	30 days	60 days
Nitrate reductase	T_N/S_	0.54 + 0.02 ^a^	0.54 + 0.00 ^a^	0.54 + 0.01 ^a^
	T_NaCl_	0.49 + 0.01 ^b^	0.51 + 0.00 ^b^	0.51 + 0.01 ^b^
	CK	0.50 + 0.02 ^b^	0.51 + 0.01 ^b^	0.51 + 0.01 ^b^
Nitrite reductase	T_N/S_	1.53 + 0.00 ^a^	1.53 + 0.01 ^a^	1.39 + 0.01 ^a^
	T_NaCl_	1.14 + 0.01 ^b^	1.12 + 0.00 ^b^	1.03 + 0.00 ^b^
	CK	1.11 + 0.01 ^c^	1.12 + 0.01 ^b^	1.01 + 0.01 ^c^

All values are presented as mean ± standard error (*n* = 5), enzyme activity is expressed in U·g^−1^, different letters in the same column under the same enzyme indicate significant differences between treatments (*p* < 0.05). Treatments: T_N/S_: NaNO_3_ + Na_2_SO_4_; T_NaCl_: NaCl; CK: control.

## Data Availability

All the data generated or analyzed during this study are included in this published article, and the data are available from the corresponding author on reasonable request.
